# Synthesis of highly functionalized β-aminocyclopentanecarboxylate stereoisomers by reductive ring opening reaction of isoxazolines

**DOI:** 10.3762/bjoc.8.10

**Published:** 2012-01-17

**Authors:** Melinda Nonn, Loránd Kiss, Reijo Sillanpää, Ferenc Fülöp

**Affiliations:** 1 Institute of Pharmaceutical Chemistry, University of Szeged, Eötvös u. 6, H-6720 Szeged, Hungary; 2 Department of Chemistry, University of Jyväskylä, FIN-40014 Jyväskylä, Finland; 3 Stereochemistry Research Group of the Hungarian Academy of Sciences, University of Szeged, Eötvös u. 6, H-6720 Szeged, Hungary

**Keywords:** amino acids, cycloaddition, functionalization, isoxazolines, reduction

## Abstract

A rapid and simple procedure was devised for the synthesis of multifunctionalized cyclic β-amino esters and γ-amino alcohols via the 1,3-dipolar cycloaddition of nitrile oxides to β-aminocyclopentenecarboxylates. The opening of the isoxazoline reductive ring to the corresponding highly functionalized 2-aminocyclopentanecarboxylates occurred stereoselectively with good yields.

## Introduction

**Figure 1:**
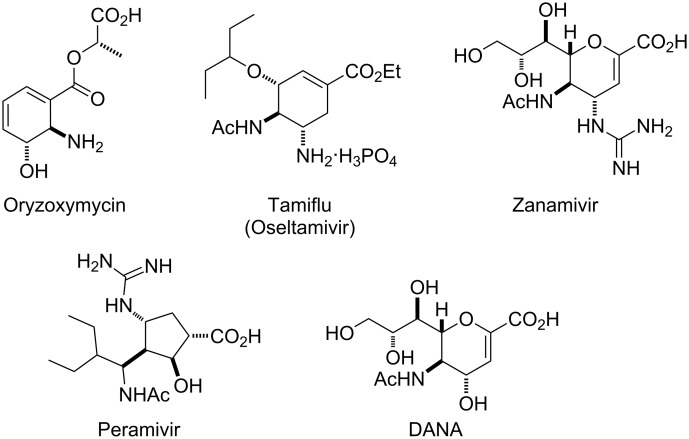
Structures of neuraminidase inhibitors.

Isoxazoline-fused amino acids are important bioactive derivatives in organic and medicinal chemistry (e.g., conformationally restricted aspartate and glutamate analogues) [1–6]. As a consequence of their ability to undergo reductive ring opening, isoxazolines are of interest as precursors for the synthesis of highly functionalized molecules such as β-hydroxyketones [7–10], amino alcohols or amino acids [11–17], etc. The multifunctionalized cyclic amino acids – e.g., the antibiotic Oryzoxymycin [18–21], the antiviral agents Tamiflu [22–33], Zanamivir and 2,3-didehydro-2-deoxy-*N*-acetylneuraminic acid (DANA) [34–38] – are bioactive derivatives of great significance for medicinal chemistry. A promising neuraminidase inhibitor, BCX-1812 (Peramivir), is currently under evaluation in clinical trials [39–45] (Figure 1). A series of Peramivir analogues has recently been investigated as potential antiviral agents [46–47].

## Results and Discussion

**Scheme 1:**
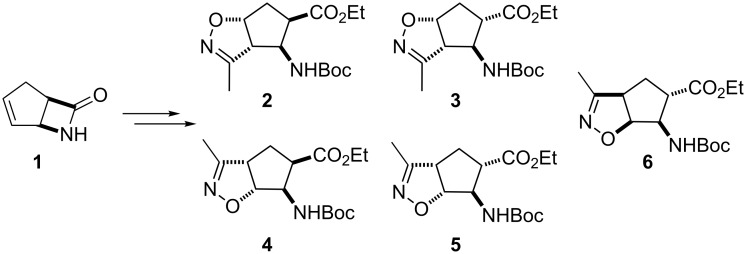
Isoxazoline-fused β-aminocyclopentanecarboxylate regio- and stereoisomers [8].

We recently reported a regio- and stereoselective procedure for the formation of a series of isoxazoline-fused cispentacin and transpentacin regio- and stereoisomers (**2–6**) from bicyclic β-lactam **1** [48–49] (Scheme 1). The syntheses consisted of a dipolar cycloaddition of nitrile oxide (generated with Boc_2_O, Et_3_N and DMAP) to the olefinic bond of *cis*-ethyl 2-aminocyclopent-3-enecarboxylate derived from **1**, during which the isoxazoline-fused amino ester regio- and stereoisomers (**2** and **4**) were formed, then separated and isolated. The cycloaddition of nitrile oxide to *trans*-ethyl 2-aminocyclopent-3-enecarboxylate under similar conditions proceeded selectively with the formation of **6**. Epimerization of **2** and **4** afforded *trans* derivatives **3** and **5** [48–49].

Since isoxazoline-functionalized molecules are excellent precursors for the construction of different functional groups through reductive ring cleavage, our recent aim was to synthesize highly functionalized β-aminocyclopentanecarboxylate regio- and stereoisomers from the earlier prepared isoxazoline-fused cispentacin and transpentacin derivatives.

A number of methods are known for the reductive opening of the isoxazoline ring: Catalytic hydrogenation or reduction with Fe in the presence of NH_4_Cl, NaBH_4_, LiAlH_4_, Raney Ni, BH_3_·THF, or SmI_2_/B(OH)_3_/H_2_O [7–17].

**Scheme 2:**
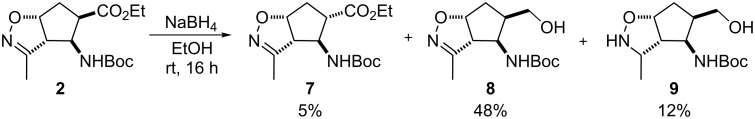
Treatment of isoxazoline-fused amino ester **2** with NaBH_4_.

For the reduction, we selected model compound **2** from earlier prepared isoxazoline-fused cispentacin stereoisomers to execute the reduction under different conditions. The isoxazoline-fused derivative was treated with the above-mentioned reducing agents. Unfortunately, neither transformation nor isoxazoline opening with ester reduction was observed. When the reduction was carried out with NaBH_4_ in EtOH, three products were obtained: The epimerized isoxazoline-fused amino carboxylate **7** and amino alcohols **8** and **9** which were separated by chromatography and isolated (Scheme 2).

**Scheme 3:**
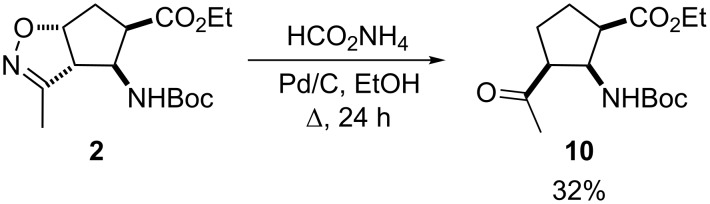
Reduction with Pd/C in the presence of HCO_2_NH_4_.

Thus, this reaction did not lead to the formation of highly functionalized isoxazoline ring-opened β-amino ester either. When ammonium formate in EtOH in the presence of Pd/C was investigated for the reduction of **2**, the ring opening resulted in carbonyl compound **10** in rather low yield through the corresponding hydroxyimine intermediate, followed by elimination and saturation (Scheme 3).

**Scheme 4:**
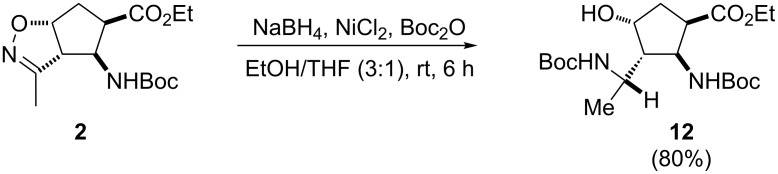
Transformation of isoxazoline-fused cispentacin stereoisomer **2** into multifunctionalized β-amino acid derivative **12**.

Combinations of NaBH_4_ (as a mild and selective reducing agent) with cobalt, nickel, iridium or rhodium halide have previously been employed for cleavage of the isoxazoline ring system, which is otherwise inert to NaBH_4_ without such metal halide additives [50]. Accordingly, we investigated the reduction of isoxazoline-fused amino ester stereoisomers **2** [48–49] with NaBH_4_ in the presence of NiCl_2_ (Scheme 4), which was found to be a suitable reducing system.

**Figure 2:**
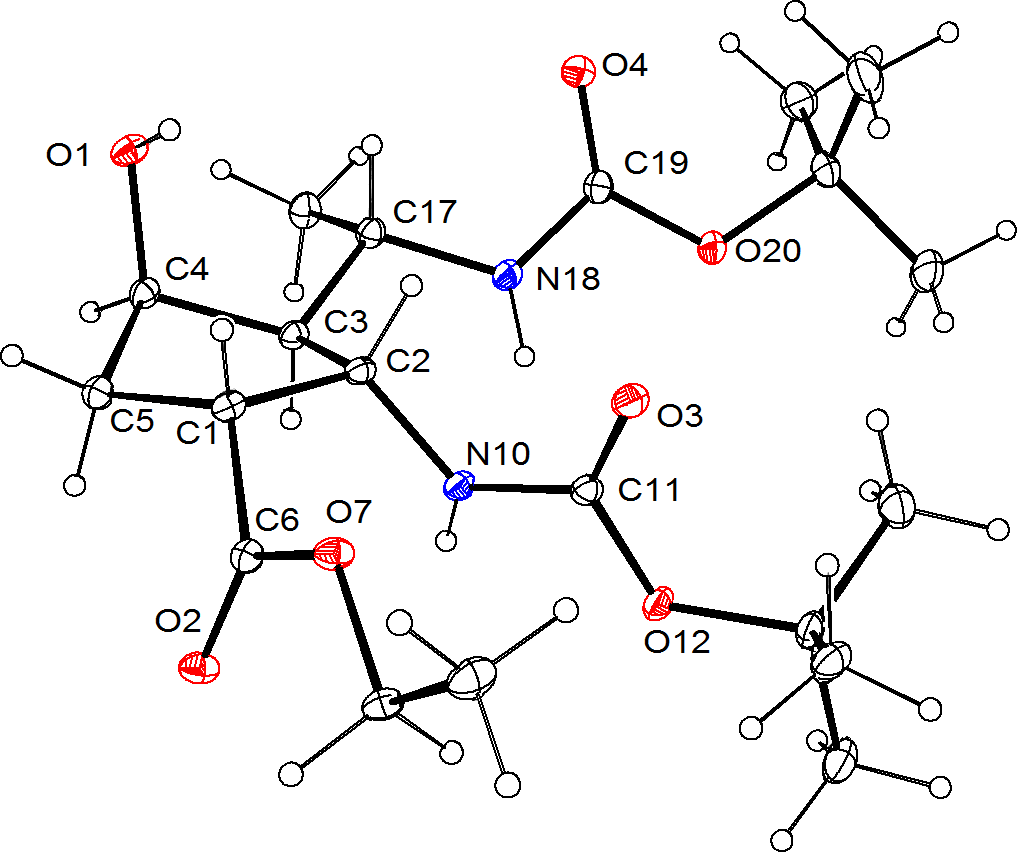
ORTEP diagram of **12** showing the atomic labeling scheme. The thermal ellipsoids are drawn at the 20% probability level.

The reduction carried out by adding NaBH_4_ to a mixture of NiCl_2_ and isoxazoline derivative **2** in EtOH/H_2_O, followed by amino group protection with Boc_2_O, selectively afforded only isoxazoline-opened product **12** as a single diastereomer in good yield. The reaction was exothermic and deposited a black granular precipitate, reflecting the presence of metal boride. The product was purified by column chromatography and the structure of **12** was certified by X-ray analysis (Figure 2).

The isoxazoline opening occurred with the formation of a new stereocenter at a one-carbon distance from C-3. In accordance with earlier results [39–47], the hydrogenation of the isoxazoline proceeded through hydrogen attack from the carbamate side (*cis* to –NHBoc) of the cyclopentane skeleton. This was confirmed by X-ray analysis of **12**.

**Scheme 5:**
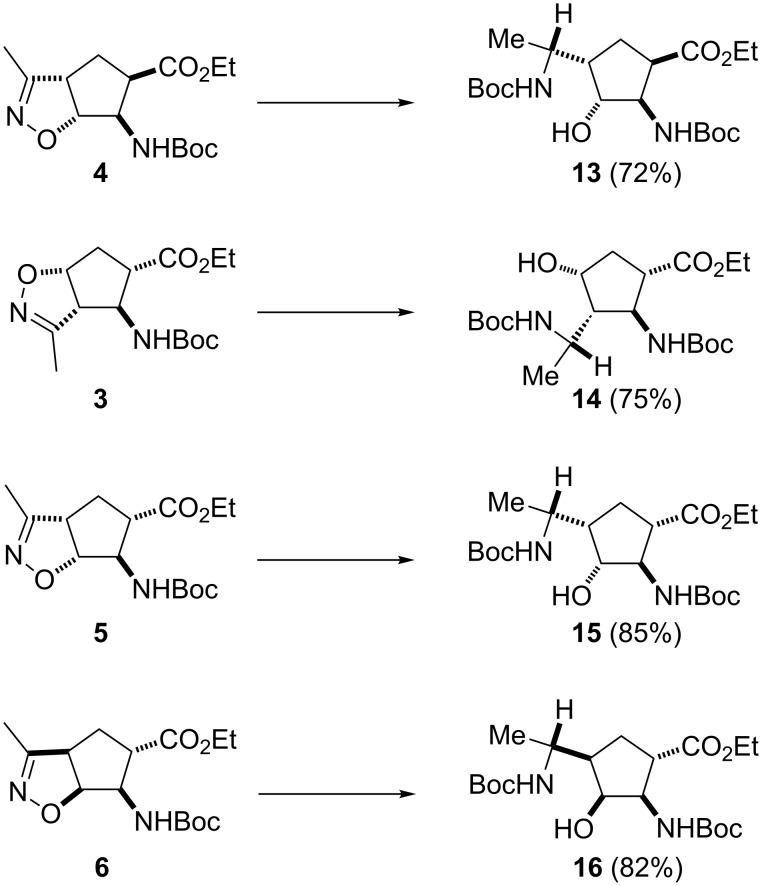
Synthesis of multifunctionalized β-amino acid derivatives **13**–**16.** Reaction conditions: NaBH_4_, NiCl_2_, Boc_2_O, EtOH/H_2_O, rt, 6 h.

In order to increase the number of multifunctionalized amino ester stereoisomers, we next examined the reductions of isoxazoline-fused cispentacin and transpentacin stereoisomers (**3**–**6**) [49]. Reactions were carried out similarly with NaBH_4_ in the presence of NiCl_2_ in EtOH/H_2_O and led selectively to the corresponding multifunctionalized amino esters **13**–**16** in good yields (Scheme 5) as single diastereoisomers.

## Conclusion

The present work has furnished a facile and efficient stereoselective reduction of isoxazoline-fused cyclic β-amino esters to multifunctionalized 2-aminocyclopentanecarboxylates through the use of NaBH_4_/NiCl_2_ as reducing agent. As Peramivir related derivatives, highly functionalized cyclic amino esters may be regarded as promising bioactive compounds.

## Experimental

The chemicals were purchased from Aldrich. The solvents were used as received from the supplier. Melting points were determined with a Kofler apparatus. NMR spectra were recorded on a Bruker DRX 400 MHz spectrometer in deuterated DMSO or CDCl_3_. Chemical shifts are expressed in ppm (δ) from the signal of internal tetramethylsilane. Mass spectra were recorded on a Finnigan MAT 95S spectrometer. Elemental analyses were recorded on a Perkin-Elmer CHNS-2400 Ser II Elemental Analyzer. FTIR spectra were recorded on a Perkin-Elmer Spectrum 100 instrument. Cycloadducts **2**–**6** were synthesized according to previously published procedures [8].

### General procedure for the synthesis of compounds **8** and **9**

To a solution of izoxazoline-fused β-aminocyclopentanecarboxylate **2** (0.96 mmol) in dry EtOH (15 mL) NaBH_4_ (2.88 mmol) was added and the reaction mixture was stirred under reflux for 16 h. The reaction was quenched by the addition of H_2_O (10 mL) and then, the mixture was concentrated under reduced pressure. The reaction mixture was diluted with H_2_O (20 mL), washed with EtOAc (3 × 15 mL), dried (Na_2_SO_4_) and concentrated under reduced pressure. The crude residue was purified by column chromatography on silica gel (*n*-hexane/EtOAc) giving **8** and **9**.

### General procedure for the synthesis of **10**

To a stirred solution of isoxazoline-fused β-aminocyclopentanecarboxylate **2** (1.6 mmol) in dry EtOH (15 mL), HCOONH_4_ (3.2 mmol) and Pd/C (0.10 g) were added and the reaction mixture was stirred under reflux for 24 h. The mixture was filtered through a celite pad and the filtrate was evaporated in vacuo. The crude residue was diluted with EtOAc (30 mL), washed with H_2_O (3 × 15 mL), dried over Na_2_SO_4_ and concentrated under reduced pressure. The residue was purified by column chromatography on silica gel (*n*-hexane/EtOAc), giving **10**.

### General procedure for isoxazoline ring opening

To a stirred solution of isoxazoline-fused β-aminocyclopentanecarboxylates **2**–**6** (0.96 mmol) in 8 mL of EtOH/THF (v:v = 3:1), NiCl_2_ (1.92 mmol) and Boc_2_O (1.92 mmol) were added. After stirring for 10 min, NaBH_4_ (1.92 mmol) was added in portions. The reaction mixture was further stirred for 6 h at room temperature and the reaction was quenched by the addition of H_2_O (5 mL). The reaction mixture was filtered through a celite pad and the filtrate was evaporated in vacuo. The crude residue was diluted with EtOAc (30 mL), washed with H_2_O (3 × 15 mL), dried over Na_2_SO_4_, and concentrated under reduced pressure. The residue was purified by column chromatography on silica gel (*n*-hexane/EtOAc), giving the corresponding reduced product.

*tert*-Butyl (3aR*,4R*,5R*,6aR*)-[5-(hydroxymethyl)-3-methyl-4,5,6,6a-tetrahydro-3a*H*-cyclopenta[*d*]isoxazol-4-yl]carbamate (8): Light-yellow oil; yield 48% (124 mg); *R*_f_ 0.35 (*n*-hexane/EtOAc); IR (KBr) ν/cm^–1^: 3344, 3265, 2979, 1678, 1563, 1184; ^1^H NMR (400 MHz, CDCl_3_) δ 1.45 (s, 3H, CH_3_), 1.56 (s, 9H, CH_3_), 1.65–1.72 (m, 2H, CH_2_), 2.19–2.25 (m, 1H, H-5), 2.75–2.81 (m, 1H, H-3a), 3.19–3.25 (m, 1H, H-6a), 3.59–3.71 (m, 1H, H-4), 3.63–3.72 (m, 2H, CH_2_), 5.42 (br s, 1H, N-H), OH group not observed – exchanged; ^13^C NMR (100 MHz, CDCl_3_) δ 16.0, 28.6, 30.2, 32.5, 43.0, 44.4, 59.2, 63.9, 78.0, 155.2, 155.6; MS (ESI) *m*/*z*: 293 [M + Na]^+^; Anal. calcd for C_13_H_22_N_2_O_4_: C, 57.76; H, 8.20; N, 10.36; found: C, 57.60; H, 8.07; N, 10.23.

*tert*-Butyl (3S*,3aR*,4R*,5R*,6aR*)-[5-(hydroxymethyl)-3-methylhexahydro-2*H*-cyclopenta[*d*]isoxazol-4-yl]carbamate (9): Colorless oil; yield 12% (31 mg); *R*_f_ 0.29 (*n*-hexane/EtOAc); IR (KBr) ν/cm^–1^: 3460, 3331, 2978, 1683, 1531, 1174; ^1^H NMR (400 MHz, CDCl_3_) δ 0.98–1.05 (m, 3H, CH_3_), 1.36 (s, 9H, CH_3_), 1.55–1.75 (m, 2H, CH_2_), 2.22–2.27 (m, 1H, H-5), 2.38–2.47 (m, 1H, H-3a), 2.78–2.86 (m, 1H, H-3), 3.17–3.24 (m, 1H, H-6a), 3.59–3.69 (m, 1H, H-4), 3.36–3.68 (m, 2H, CH_2_), 5.32 (br s, 1H, N-H), 6.12 (br s, 1H, N-H), OH group not observed – exchanged; ^13^C NMR (100 MHz, CDCl_3_) δ 15.0, 27.1, 29.0, 35.8, 42.4, 51.7, 57.2, 62.6, 77.4, 80.4, 155.6; MS (ESI) *m*/*z*: 295 [M + Na]^+^; Anal. calcd for C_13_H_24_N_2_O_4_: C, 57.33; H, 8.88; N, 10.29; found: C, 57.20; H, 8.71; N, 10.42.

Ethyl (1R*,2S*,3S*)-3-acetyl-2-(*tert*-butoxycarbonylamino)cyclopentanecarboxylate (10): White solid; yield 32% (153 mg); mp 109–110 °C; *R*_f_ 0.62 (*n*-hexane/EtOAc); IR (KBr) ν/cm^–1^: 3354, 2978, 1716, 1684, 1531, 1171; ^1^H NMR (400 MHz, CDCl_3_) δ 1.29 (t, *J* = 7.54 Hz, 3H, CH_3_), 1.41 (s, 9H, CH_3_), 1.59–1.71 (m, 2H, CH_2_), 1.74–1.95 (m, 2H, CH_2_), 2.05 (s, 3H, CH_3_), 2.83–2.97 (m, 1H, H-1), 3.01–3.15 (m, 1H, H-3), 4.18–4.29 (m, 2H, OCH_2_), 4.31–4.44 (m, 1H, H-2), 5.76 (br s, 1H, N-H); ^13^C NMR (100 MHz, CDCl_3_) δ 13.98, 20.05, 25.76, 29.31, 31.21, 43.97, 46.01, 52.70, 82.01, 155.67, 176.01, 206.52; MS (ESI) *m*/*z*: 322 [M + Na]^+^; Anal. calcd for C_15_H_25_NO_5_: C, 60.18; H, 8.42; N, 4.68; found: C, 60.05; H, 8.35; N, 4.54.

Ethyl (1R*,2S*,3S*,4R*)-2-(*tert*-butoxycarbonyl)-3-((S*)-1-(*tert*-butoxycarbonyl)ethyl)-4-hydroxycyclopentanecarboxylate (12): White solid; yield 80% (320 mg); mp 120–121 °C; *R*_f_ 0.22 (*n*-hexane/EtOAc 1:1); IR (KBr) ν/cm^–1^: 3457, 3348, 2982, 1720, 1698, 1531, 1160; ^1^H NMR (400 MHz, DMSO) δ 0.96 (t, *J* = 7.34 Hz, 3H, CH_3_), 1.27–1.33 (m, 3H, CH_3_), 1.45–1.50 (m, 18H, CH_3_), 1.94–2.02 (m, 2H, CH_2_), 2.07–2.16 (m, 1H, H-4), 3.30–3.39 (m, 1H, H-1), 3.80–3.89 (m, 1H, CH), 4.13–4.23 (m, 2H, OCH_2_), 4.24–4.30 (m, 1H, H-2), 4.44–4.56 (m, 1H, H-3), 5.28–5.35 (m, 1H, NH), 5.61–5.72 (m, 1H, NH), OH group not observed – exchanged; ^13^C NMR (100 MHz, CDCl_3_) δ 11.7, 14.6, 28.8, 28.9, 29.3, 30.1, 31.8, 37.3, 44.6, 51.1, 54.6, 61.2, 73.7, 80.1, 80.4, 155.0, 156.5, 172.0; MS (ESI) *m*/*z*: 418 [M + 2H]^+^; Anal. calcd for C_20_H_36_N_2_O_7_: C, 57.67; H, 8.71; N, 6.73; found: C, 57,44; H, 8.86; N, 6.58.

Ethyl (1R*,2R*,3R*,4S*)-2-(*tert*-butoxycarbonyl)-4-((R*)-1-(*tert*-butoxycarbonyl)ethyl)-3-hydroxycyclopentanecarboxylate (13): White solid; yield 72% (288 mg); mp 129–130 °C; *R*_f_ 0.59 (*n*-hexane/EtOAc 1:1); IR (KBr) ν/cm^–1^: 3479, 3347, 3353, 1725, 1685, 1662, 1531, 1163; ^1^H NMR (400 MHz, CDCl_3_) δ 1.17–1.29 (m, 6H, CH_3_), 1.40–1.46 (m, 18H, CH_3_), 1.79–1.91 (m, 1H, CH_2_), 2.05–2.19 (m, 2H, CH_2_, H-1), 3.26–3.34 (m, 1H, H-4), 3.86–4.01 (m, 2H, H-2, CH), 4.08–4.19 (m, 3H, OCH_2_, H-3), 4.53 (br s, 1H, N-H), 5.05 (br s, 1H, N-H), OH group not observed – exchanged; ^13^C NMR (100 MHz, CDCl_3_) δ 14.6, 21.1, 28.4, 28.7, 28.8, 44.0, 46.6, 60.5, 61.2, 67.5, 77.6, 80.2, 86.4, 156.1, 156.4, 174.8; MS (ESI) *m*/*z*: 418 [M + 2H]^+^; Anal. calcd for C_20_H_36_N_2_O_7_: C, 57.67; H, 8.71; N, 6.73; found: C, 57.50; H, 8.98; N, 6.39.

Ethyl (1S*,2S*,3S*,4R*)-2-(*tert*-butoxycarbonyl)-3-((S*)-1-(*tert*-butoxycarbonyl)ethyl)-4-hydroxycyclopentanecarboxylate (14): White solid; yield 75% (300 mg); mp 144–145 °C; *R*_f_ 0.3 (*n*-hexane/EtOAc 1:1); IR (KBr) ν/cm^–1^: 3420, 3363, 2980, 1692, 1537, 1185; ^1^H NMR (400 MHz, CDCl_3_) δ 1.26–1.33 (m, 6H, CH_3_), 1.43–1.48 (m, 18H, CH_3_), 1.82–1.93 (m, 1H, CH_2_), 1.98–2.15 (m, 1H, H-1), 2.24–2.36 (m, 1H, CH_2_), 2.76–2.89 (m, 1H, H-3), 3.58–3.72 (m, 1H, H-4), 3.93–4.05 (m, 1H, H-2), 4.15–4.25 (m, 3H, OCH_2_, CH), 4.87 (br s, 1H, N-H), 5.09 (br s, 1H, N-H), OH group not observed – exchanged; ^13^C NMR (100 MHz, CDCl_3_) δ 14.5, 21.4, 28.8, 28.9, 35.9, 45.7, 49.1, 52.3, 54.5, 58.3, 73.4, 80.1, 152.5, 156.8, 172.6; MS (ESI) *m*/*z*: 418 [M + 2H]^+^; Anal. calcd for C_20_H_36_N_2_O_7_: C, 57.67; H, 8.71; N, 6.73; found: C, 57.41; H, 8.37; N, 6.59.

Ethyl (1S*,2R*,3R*,4S*)-2-(*tert*-butoxycarbonyl)-4-((R*)-1-(*tert*-butoxycarbonyl)ethyl)-3-hydroxycyclopentanecarboxylate (15): White solid; yield 85% (340 mg); mp 141–142 °C; *R*_f_ 0.46 (*n*-hexane/EtOAc 1:1); IR (KBr) ν/cm^–1^: 3426, 3378, 3333, 2979, 1688, 1718, 1703, 1522, 1176; ^1^H NMR (400 MHz, CDCl_3_) δ 1.21–1.30 (m, 6H, CH_3_), 1.40–1.46 (m, 18H, CH_3_), 1.84–1.97 (m, 2H, CH_2_, H-4), 2.03–2.20 (m, 2H, CH_2_, H-1), 2.54 (q, *J* = 9.10 Hz, 1H, H-2,), 3.73–3.82 (m, 1H, H-3), 3.87–4.04 (m, 2H, N-H, CH), 4.10–4.22 (m, 2H, OCH_2_), 4.83 (br s, 1H, N-H), OH group not observed – exchanged; ^13^C NMR (100 MHz, CDCl_3_) δ 14.1, 20.0, 27.5, 28.7, 28.8, 45.6, 46.1, 46.8, 60.9, 62.5, 78.1, 80.1, 80.3, 154.0, 156.4, 174.6; MS (ESI) *m*/*z*: 418 [M + 2H]^+^; Anal. calcd for C_20_H_36_N_2_O_7_: C, 57.67; H, 8.71; N, 6.73; found: C, 57.91; H, 8.46; N, 6.58.

Ethyl (1S*,2R*,3S*,4R*)-2-(*tert*-butoxycarbonyl)-4-((S*)-1-(*tert*-butoxycarbonyl)ethyl)-3-hydroxycyclopentanecarboxylate (16): White solid; yield 82% (328 mg); mp 166–167 °C; *R*_f_ 0.32 (*n*-hexane/EtOAc 1:1); IR (KBr) ν/cm^–1^: 3485, 3368, 3353, 2975, 1733, 1681, 1667, 1533, 1167; ^1^H NMR (400 MHz, CDCl_3_) δ 1.17–1.31 (m, 6H, CH_3_), 1.38–1.46 (m, 18H, CH_3_), 1.79–2.15 (m, 3H, CH_2_, H-1, H-4), 2.72–2.87 (m, 1H, CH_2_), 3.77–4.03 (m, 1H, CH), 4.06–4.23 (m, 4H, H-2, H-3, OCH_2_), 4.37–4.48 (m, 1H, N-H), 4.88 (br s, 1H, N-H), OH group not observed – exchanged; ^13^C NMR (100 MHz, CDCl_3_) 14.6, 21.6, 28.7, 28.8, 47.2, 49.0, 59.9, 61.2, 61.6, 69.4, 74.7, 80.0, 85.9, 117.5, 156.1, 158.8, 171.3; MS (ESI) *m*/*z*: 418 [M + 2H]^+^; Anal. calcd for C_20_H_36_N_2_O_7_: C, 57.67; H, 8.71; N, 6.73; found: C, 57.43; H, 8.40; N, 6.95.

**X-ray crystallographic study of 12:** Crystallographic data were collected at 123 K with a Nonius-Kappa CCD area detector diffractometer, using graphite-monochromatized Mo K_a_ radiation (λ = 0.71073 Å) as reported earlier [51]. **Crystal data for 12**, C_20_H_36_N_2_O_7_, *M_r_* = 416.51, triclinic, space group *P*−1 (no. 2), *a* = 9.3765(2), *b =* 13.7078(4), *c* = 18.7792(4) Å, α = 96.609(2), β = 95.261(1), γ = 100.965(1), *V* = 2337.9(1) Å^3^, *T* = 123 K, *Z* = 4, μ(Mo K_α_) = 0.089 mm^–1^, 9120 unique reflections (*R_int_* = 0.034) which were used in calculations. The final *R1* (for the data with *F^2^* > 2δ*(F^2^)* was 0.042 and *wR2*(*F^2^*) (all data) was 0.111.

The SHELXL-97 program [52] was used to solve the structure by direct methods and to perform full-matrix, least-squares refinements on *F^2^*. The unit cell of **12** contains two molecules with slightly different conformations. The CH hydrogen atoms were included at fixed distances from their host atoms with fixed displacement parameters. The NH and OH hydrogen atoms were refined isotropically. The deposition number CCDC 845835 contains the supplementary crystallographic data for this paper. These data can be obtained free of charge at http://www.ccdc.cam.ac.uk/conts/retrieving.html [or from the Cambridge Crystallographic Data Centre, 12 Union Road, Cambridge CB2 1EZ, UK; Fax: (internat.) +44-1223-336-033; Email: deposit@ccdc.cam.ac.uk].
